# qSOFA does not replace SIRS in the definition of sepsis

**DOI:** 10.1186/s13054-016-1389-z

**Published:** 2016-07-17

**Authors:** Jean-Louis Vincent, Greg S. Martin, Mitchell M. Levy

**Affiliations:** Department of Intensive Care, Erasme University Hospital, Université Libre de Bruxelles, Route de Lennik 808, 1070 Brussels, Belgium; Division of Pulmonary, Allergy and Critical Care, Emory University School of Medicine, Grady Memorial Hospital, 615 Michael Street, Suite 205, Atlanta, GA 30322 USA; Division of Pulmonary, Critical Care and Sleep Medicine, Alpert Medical School at Brown University, 593 Eddy Street, Providence, RI 02903 USA

The recently published consensus definitions for sepsis [1] have raised a lot of discussion and controversy. We had the privilege of being part of this consensus group and fully support the final definitions. We are pleased that a definition has been developed that closely reflects everyday clinical language, recognizing that sepsis is most simply described as a “bad infection” associated with some degree of organ dysfunction, as proposed earlier [2]. The article conveying the consensus definition [1] also emphasizes that sepsis is more often recognized from the associated organ dysfunction than from the more difficult to identify infection, so that sepsis can be defined as “life-threatening organ dysfunction caused by a dysregulated host response to an infection”.

The proposition of the 1992 North American consensus document [3] that sepsis be defined by a combination of the systemic inflammatory response syndrome (SIRS) and the presence of an infection raised confusion, because the SIRS criteria (especially fever, tachycardia, and altered white blood cell count) are themselves typical features of infection [3]. As the majority of infected patients will therefore meet the SIRS criteria, they would also be considered to have sepsis by this 1992 definition. This approach to defining sepsis has resulted in a dramatic increase in the number of patients diagnosed with sepsis over the years [4]; however, these patients may have less severe disease so that reported parallel reductions in mortality rates [5] may be deceptive [6]. The recent “new” definitions are not so novel, more a return to the traditional use of the term to indicate patients with a substantial and deleterious response to an infection. We doubt that this will change further over time, exactly as the meaning of other words like pneumonia, peritonitis, or meningitis has not changed.

We all agree on the fundamental importance of identifying sepsis early and of applying effective and complete treatment to minimize complications. However, the SIRS criteria were too sensitive and not sufficiently specific for this purpose. Rangel-Frausto et al. [7] reported that 68 % of patients admitted to three intensive care units (ICUs) and three general wards met the SIRS criteria; in 198 ICUs in 24 European countries, Sprung et al. [8] reported that 93 % of ICU patients had at least two SIRS criteria at some point during their ICU stay; and in a database of patients in 23 Australian and New Zealand ICUs, Dulhunty et al. [9] reported that 88.4 % of patients had at least two SIRS criteria on ICU admission. In a recent analysis of a large US database, Churpek et al. [10] reported that almost half of the 270,000 patients hospitalized on regular wards met the SIRS criteria at one time or another. Our consensus definition paper suggested the quick sequential organ failure assessment (qSOFA) as an effective way of raising suspicion of sepsis on the regular floor [1]. Evaluating all six components of the SOFA score can be time consuming, and some require laboratory measurements. By analyzing a large database of hospitalized patients, three clinical elements (hypotension, altered mentation, and tachypnea) were identified that could be used at the bedside to recognize those infected patients who are at risk of deteriorating or having a complicated course (death or ICU stay ≥ 3 days). The presence of two or more of these criteria can be used to prompt clinicians to further evaluate the patient for the presence of infection and/or organ dysfunction, to start or adapt treatment, and to consider transfer to an ICU. Importantly, this approach is designed to be an early warning system, and a patient with less than two qSOFA criteria may still raise concern. Clinical judgment should always supersede tools designed to help improve patient care, such as qSOFA.

We would like to stress that, although SIRS was part of the definition of sepsis in 1992 [3], the qSOFA is not part of the new sepsis definitions. This important difference is illustrated in Fig. 1, with panel A showing that infection and sepsis (by the 1992 definition) are virtually the same—infection without SIRS can be found, but it is relatively rare. By contrast, panel B shows that sepsis (by the new SEPSIS-3 definition) represents only a minority of cases of infection. Moreover, panel B illustrates important aspects of the sepsis definition vis-à-vis infection and qSOFA. For example, sepsis can be present without a qSOFA score ≥ 2 because different forms of organ dysfunction may be present than are assessed using the qSOFA, such as hypoxemia, renal failure, coagulopathy, or hyperbilirubinemia. In addition, a patient may have a qSOFA ≥ 2 without infection; for example, in other acute conditions, such as hypovolemia, severe heart failure, or large pulmonary embolism. Further work remains to be done to determine the predictive validity of qSOFA in such patients. Finally, infected patients may have a qSOFA ≥ 2 and not be septic because the degree of hypotension, tachycardia, and/or altered mentation needed to fulfill qSOFA criteria is not the same as that needed to meet the SOFA organ dysfunction criteria necessary for a diagnosis of sepsis; the qSOFA criteria are thus clinically valuable but imperfect markers of sepsis. Nevertheless, in an analysis of a database of more than 74,000 patients, Seymour et al. [11] recently reported that 75 % of patients with suspected infection who had two or more qSOFA points also had at least two SOFA points.

**Fig. 1:**
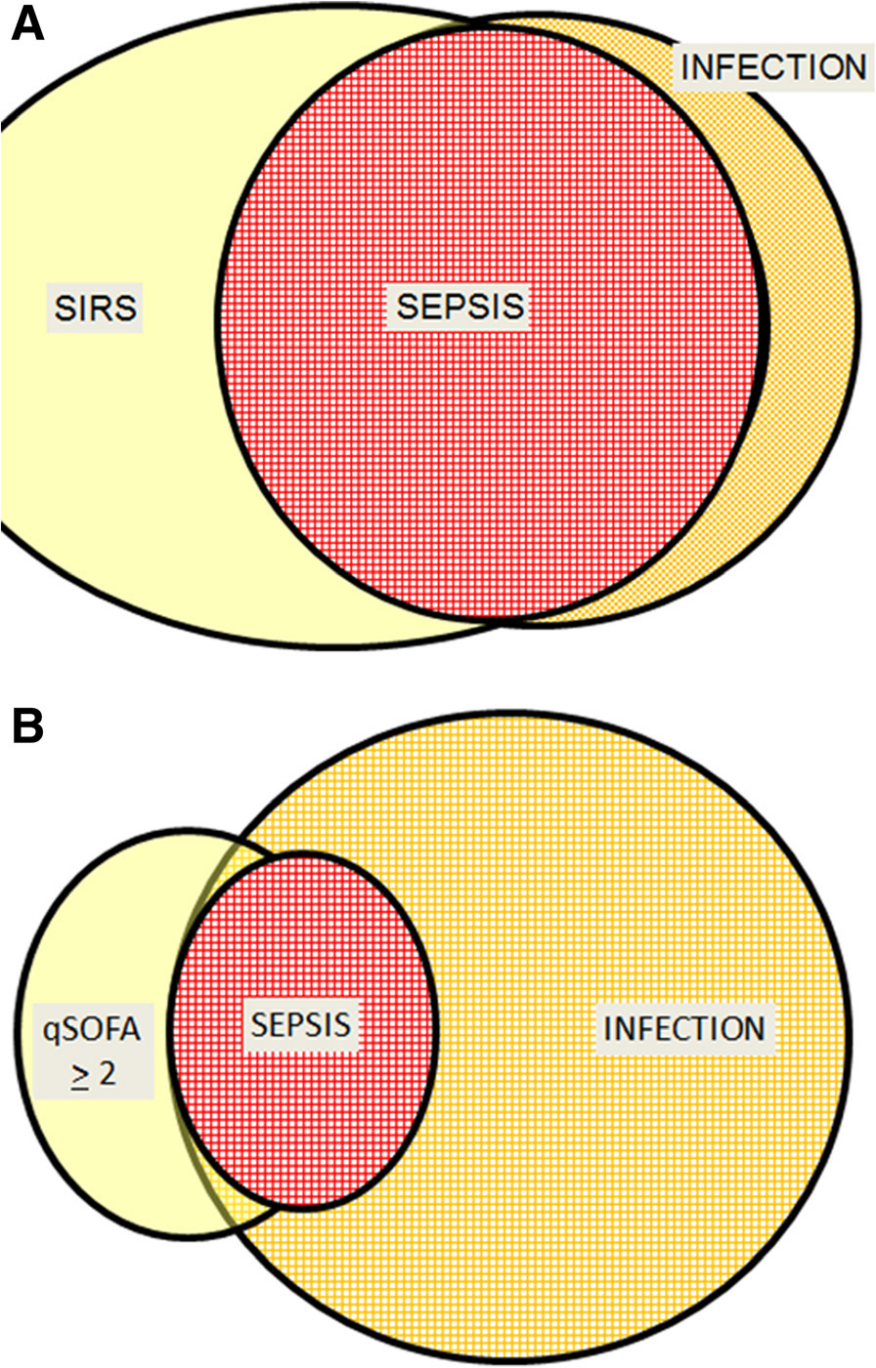
Schematic representation illustrating **a** the almost complete overlap of sepsis and infection when the SIRS criteria of the 1992 criteria [3] are used and **b** the differences between qSOFA and sepsis. *qSOFA* quick sequential organ failure assessment, *SIRS* systemic inflammatory response syndrome

We hope this editorial will clarify that the qSOFA is meant to be used to raise suspicion of sepsis and prompt further action—it is not a replacement for SIRS and is not part of the definition of sepsis.

## Abbreviations

ICU, intensive care unit; qSOFA, quick sequential organ failure assessment; SIRS, systemic inflammatory response syndrome; SOFA, sequential organ failure assessment.
